# SW Neuronet – neuroscience for psychiatrists update day

**DOI:** 10.1192/bjo.2021.375

**Published:** 2021-06-18

**Authors:** Gabrielle Churchhouse, Lindsey Sinclair, Simon George Morris

**Affiliations:** 1 Gloucestershire Health and Care NHS Foundation Trust; 2 University of Bristol, Avon and Wiltshire Mental Health Partnership NHS Trust; 3 Avon and Wiltshire Mental Health Partnership NHS Trust

## Abstract

**Aims:**

The Royal College of Psychiatrists Neuroscience Project was established to promote greater integration of modern neuroscience into psychiatric training and practice. Regional “Neuronets” are being established to develop local learning opportunities. As the Southwest Neuronet, we sought to establish a high quality and sustainable regional educational event promoting modern neuroscience in psychiatry.

**Method:**

We developed and ran two events in collaboration with the Neuroscience Project, a whole day in-person event in September 2019 and a half day online event in January 2021. Attendees were invited from the Southwest with the latter event being shared more widely through other “Neuronets”. Both featured talks by leading experts in the neuroscience of psychiatry. The first was themed around “Neuroscience from the lab to the clinic”, building on basic research methodologies to their applications in clinical psychiatry. Our pandemic era online event, “Neuroscience of psychosis”, was structured around an evolving clinical case. Both featured interactive elements using audience polling technology to gather views and collate questions. Feedback was gathered through an online survey with individual session ratings and event ratings.

**Result:**

154 people attended the in-person event from across the South West Division. This included psychiatry trainees, consultants and a small number of other mental health professionals. 382 people signed up to our online event with 262 attending live and others watching recorded sessions. Feedback response rates were 42% and 33% respectively. Feedback on the practical arrangements was highly positive, particularly highlighting pre-event communication. Attendees valued the high calibre of speakers and particularly rated topics of psychiatric genetics, novel antidepressants, and autoimmune psychosis. Environmental sustainability was a prominent theme in our first event with support for our paperless approach but highlighted further potential to reduce waste associated with catering. Overall, attendees valued the opportunity to build on knowledge of basic research techniques but also wished to see greater focus on clinical applications of neuroscience, which we had responded to with the inclusion of a clinical case to frame our online event.

**Conclusion:**

These events provide a prototype for low-cost regional neuroscience in psychiatry education events, in-person or online. Sustainability in terms of cost, human resources for organisation, and environmental impact are all important considerations for such events. We plan to continue to run these annually, forming part of the legacy of the Neuroscience Project. In line with feedback received, we seek to maximise the clinical relevance but also share novel research techniques encountered in the literature.

